# Development of a New Resequencing Pathogen Microarray Based Assay for Detection of Broad-Spectrum Respiratory Tract Viruses in Patients with Community-Acquired Pneumonia

**DOI:** 10.1371/journal.pone.0075704

**Published:** 2013-09-27

**Authors:** Hongwei Shen, Weixian Shi, Ji Wang, Miao Wang, Jin Li, Chen Zhang, Kai Nie, Mengjie Yang, Yi Zhang, Aihua Li, Wenjie Tan, Xuejun Ma

**Affiliations:** 1 Key Laboratory of Medical Virology, Ministry of Health, National Institute for Viral Disease Control and Prevention, Chinese Center for Disease Control and Prevention, Beijing, China; 2 Futian District Center for Disease Control and Prevention, Shenzhen, Guangdong, China; 3 Institute for Infectious Disease and Endemic Disease Control, Beijing Center for Disease Control and Prevention, Beijing, China; 4 Institute for Immunization and Prevention, Beijing Center for Disease Control and Prevention, Beijing, China; Naval Research Laboratory, United States of America

## Abstract

A Resequencing Pathogen Microarray (RPM) is a single, highly multiplexed assay for detecting and differentiating similarly related pathogens by using closely overlapping probe sets to determine a target organism’s nucleotide sequence. In this study, a new RPM (RPM-IVDC1) that consisted of 224-bp detector tiles corresponding to 9 influenza A subtypes, 11 rhinoviruses, 28 enteroviruses and 38 other respiratory viruses was developed and optimized to provide individual and simultaneous detection sensitivities ranging from 15 to 750 genomic copies for 16 common respiratory pathogens. A total of 110 consecutive patients with community-acquired pneumonia (CAP) admitted to 5 district general hospitals in Beijing during a 1-year period were assessed using the new assay. Among the children (under age 5) and adult patients (above age 18), respiratory syncytial virus (RSV) and rhinovirus (RV) were the most common etiological agents, respectively, which is consistent with reference assays. Atypical pathogens that may cause CAP-like illness, including rubella virus, measles virus, influenza type C virus, human herpesvirus (HHV) were also detected. The results show the capability of RPM-IVDC1 for the accurate detection and identification of multiple virus types, which may be of significant use in epidemic surveillance and outbreak investigations of atypical pathogens.

## Introduction

Pneumonia is a common clinical entity, particularly among the elderly [1]. In addition, among young children in many developing countries, community-acquired pneumonia (CAP) is responsible for a significant number of deaths [2]. More than 2 million children under age 5 are killed by pneumonia every year worldwide—more than AIDS, malaria, and measles combined [3]. For each child who dies of pneumonia in a developed country, more than 2,000 die in developing countries [4,5].

Identification of the etiological agent causing CAP is critical for defining proper treatment and the introduction of preventive measures. Although a limited number of pathogens are responsible for the vast majority of cases, a wide variety of etiological agents may cause CAP [6]. Many studies have been conducted to investigate the bacterial etiology of CAP [7,8]. In contrast to the vast amount of knowledge we have about bacterial agents, the viral etiology of CAP has not been paid an equivalent attention.

Respiratory syncytial virus (RSV), influenza type A or B virus (FluA or FluB), rhinovirus (RV), parainfluenza virus type 1-3 (PIV1-PIV3), adenovirus (AdV), human metapneumovirus (hMPV) and enterovirus (EV) have been analyzed in most CAP studies, and it has been shown that RSV and RV are the most common agents associated with CAP in children [9-11], while RV, coronaviruses (CoV-OC43, CoV-229E) are frequently identified in adult patients [12]. The severe acute respiratory syndrome coronavirus (SARS-CoV), the H5N1 strain of influenza A virus and adenovirus serotype 14 have been also received focused attention as causes of severe lower respiratory tract infections [6]. Although the common microbial agents accounted for majority CAP have been established, about 19%-45% of cases of the disease are still caused by unknown pathogens [1].

Viral culture and direct fluorescent antigen detection (DFA) are the traditional gold standard diagnostic tests for respiratory viral pathogens [13], yet these assays can be labor-intensive and time-consuming. With the advantage of detecting multiple pathogens simultaneously, various multiplex PCRs have been developed and optimized to be performed on lower respiratory tract samples, offering the opportunity for increased sensitivity and specificity of the diagnosis and improved outcomes [14,15]. These multiplexed methods are being broadly used to detect the common etiological agents of CAP. However, there is new information on the incidence of atypical pathogens and of pathogens in cases of severe CAP and in CAP in the elderly [1], for which the multiplexed assays have not been used to detect.

As so many pathogens can cause pneumonia with similar symptoms, a method for the unambiguous detection of a broad range of microbial agents simultaneously is highly in demand. Resequencing Pathogen Microarray (RPM) is a single, highly multiplexed and simultaneous differential diagnostic assay [16]. Furthermore, the sequence information produced by RPM enables achieving high-resolution pathogen identification and near-neighbor discrimination [17,18]. The advantages over competing technologies make RPM highly suitable for outbreak investigations caused by atypical pathogen or uncommon pathogens.

In this study, 110 nasopharyngeal aspirates were collected to assess the broad-range respiratory tract viral agents detection ability of a new RPM (RPM-IVDC1) designed by TessArae LLC and the Institute of Viral Disease Control and Prevention (IVDC), Chinese Center for Disease Control and Prevention (CCDC). The RPM-IVDC1 assay could sequence 47,974 bp of both strands of targeted gene sequences, distributed across 183 detector tiles (each 224-bp in length), representing 86 types/subtypes of viral pathogens and 21 other respiratory tract pathogens that cause respiratory infection. The viruses targeted by the RPM-IVDC1 assay are identified in Material S1, consisting of 9 influenza A subtypes, 11 rhinoviruses, 28 enteroviruses and 38 other viruses.

## Materials and Methods

### Specimen Collection and Sample Processing

Nasopharyngeal aspirates (NPAs) were consecutively collected by the Beijing Center for Disease Control and Prevention (CDC) from patients with a diagnosis of CAP in five Beijing district general hospitals from January 2011 to December 2011. Patients were included if they had at least one of following: symptoms of acute infection and respiratory tract disease manifestations, pneumonia confirmed by chest radiography, or clinical diagnosis of pneumonia. Patients with fever (≧37.5°C) or chills or changes in distribution of white blood cell (WBC) were identified as acute infection. Symptoms of respiratory tract disease may include at least one of the following: cough with or without expectoration, dyspnea, chest discomfort, or auscultory lung findings. A total of 110 patients (62 of whom were male) meeting the criteria, aged from 1 month to 96 years old, were enrolled in this study (Table 1). For each patient, a standard questionnaire including demographic data, radiological result and clinical signs was filled out. The NPAs were kept at -70°C until further study. All aspects of the study were performed in accordance with national ethics regulations and approved by the Institutional Review Boards of the Centre for Disease Control and Prevention of China, as well as the Ethics Committee of the Beijing CDC. Participants received information on the study’s purpose and of their right to keep information confidential. Written consent was obtained from each adult participant and children’s parents or their guardians.

**Table 1:** The epidemiological characteristics of specimens (n=110) in this study.

Category	Subcatergory	No. (%)
Sex	Female	62 (56.4)
	Male	48 (43.6)
Age	0-5	22 (20.0)
	6-17	20 (18.2)
	18-96	68 (61.8)

Nucleic acid was extracted and purified from the NPAs using the MasterPure Complete DNA and RNA purification kit (Epicenter Technologies, Madison, WI) according to the manufacturer’s recommended protocols. Purified nucleic acid was frozen at -80°C in 15-µl aliquots.

### Internal Controls

Two *Arabidopsis thaliana* plant genes, corresponding to triosphosphate isomerase (TIM) and NAC domain-containing protein 21/22 (NAC1), were chosen as internal controls for reverse transcription (RT) and multiplex PCR. DNA was obtained from 10-day plantlet, which was kindly provided by Chinese Academy of Science. TIM (TIM-1, TIM-2, corresponding to different fragments of TIM) and NAC1 (NAC1-1, NAC1-2, NAC1-3, corresponding to different fragments of NAC1) were amplified from the whole genome and purified using a QIAquick PCR purification kit (QIAGEN, Hilden, Germany). The purified products were ligated to pGEM-T vector (Promega, Madison, WI) and the recombinant plasmids were transformed into *E. coli*. The recombinant plasmid of TIM was linearized with *Spe*I and *in vitro* transcribed from the T7 promoter using Ribo-MAX™ large scale RNA Production System T7 (Promega, Madison, WI). A total of 10,000 copies of *in vitro* transcribed TIM RNA and 1,000 copies of NAC1 plasmid were used as internal controls for checking the amplification efficiency of RT-PCR and multiplex PCR as well as the presence of inhibitors in the specimens [19].

### Primer design and multiplex RT-PCR amplification

The gene-specific primer pairs were designed by TessArae LLC and the Institute of Viral Disease Control and Prevention (IVDC), CCDC with similar annealing temperatures to assure the approximate amplification efficiency. To achieve good sensitivity and specificity, one or more conserved genes of each pathogen were chosen as targets. The uniqueness of all primers was checked using a full search of the GenBank database with the BLAST program [19]. The method of Tania Tabone, et al. [20] was adapted to reduce the intra-primer interaction and to further ensure the similar amplification efficiency by adding a linker sequence of 18 bases (forward universal tag sequence, UTS-F) and 19 bases (reverse universal tag sequence, UTS-R) to the 5’ ends of forward and reverse primers, respectively. To further minimize the possibility of potential of primer dimers and to help ensure sufficient amplifications, the primers were divided into four independent reactions, which simplified primer design and optimization.

RT reactions were performed in 20-µl volume containing 500µM each dATP, dTTP, dGTP and dCTP, 50ng of random hexamers, 1x RT buffer, 5mM MgCl_2_, 10mM DTT, 40U of RNaseOUT, 200U of SuperScript III (Invitrogen Life Technologies, Carlsbad, CA), 10,000 copies of *in vitro* transcribed TIM RNA and 2µl of the extracted clinical specimen. Reactions were carried out in a Px2 Thermo Cycler (Thermo Electron Corp, Vantaa, Finland) using the manufacturer’s recommended protocol.

**Table 2:** Strain identification by RPM-IVDC1 detection of viruses causing respiratory infections.

Virus^a^	identification by RPM-IVDC1 (GenBank accession no.)^b^	C3 score (detector gene)^c^
AdV	AdV B1	89 (E1A)
AdV	AdV B2	83(E1A)
AdV	AdV C	57.5 (E1A)
AdV	AdV DA	52.5 (E1A)
H5N1	H5N1	93.5 (M), 52.5 (HA)
FluB	FluB	75 (M)
hMPV	hMPV (HM197719.1)	100 (NCP), 97 (M)
CoV-OC43	CoV-OC43 (AY585229.1)	82 (spike), 60 (NP)
CoV-229E	CoV-229E	96.5 (NP), 92 (spike)
CoV-HKU1	CoV-HKU1	98 (NP), 80 (spike)
RSVA	RSVA (DQ780565.1)	97.5 (NP), 78.5(GP)
RSVB	RSVB (AF013254.1)	97 (NP), 79.5(GP)
PIV1	PIV1 (M80818.1)	89 (M), 71 (HN)
PIV3	PIV3 (FJ455842.2)	95.5 (M), 88 (HN)
RV	RVA	79.5 (5’UTR)
RV	RVB	78.5 (5’UTR)
RV	RVC	51.5 (5’UTR)
HBoV	HBoV	94 (VP1), 87.5 (NP1)

^a^ The validation was performed with clinical samples infected with known virus.

^b^ The BLAST search results from sequences generated from samples demonstrated that RPM-IVDC1 not only correctly identified these samples but also differentiated some pathogens.

^c^ The C3 scores from RPM-IVDC1 gene sequence detector tiles indicated that RPM-IVDC1 could accurately identify the samples when the threshold was set to C3≧50 The C3 scores from sub-threshold negative detector tile and noninterfering hybridized detector tiles were not shown in this table.

(M) matrix; (HA) hemagglutinin.

A 2-µl aliquot of RT product was subjected to each of the four different multiplex PCRs. Primer mix A contained 38 primer pairs corresponding to 38 target genes of common respiratory viruses including four AdV serotypes, four CoV species, two RSV subgroups, two hMPV subtypes, four PIV types, human bocavirus (HBoV), three influenza A subtypes, one influenza B virus, one influenza C virus, three RV serotypes, and one internal control (TIM-1) (see Table S1 in Material S1). Primer mix B contained 36 primer pairs corresponding to 32 target genes of 5 influenza A subtypes, eight RV serotypes, three PIV types, CoV-OC43 and one internal control (NAC1-1) (see Table S2 in Material S1). Primer mix C contained 36 primer pairs corresponding to 28 target genes of nine coxsackievirus A subtypes, three coxsackievirus B subtypes, ten echovirus types, three poliovirus types, three enterovirus types and two internal controls (TIM-2 and NAC1-2) (see Table S3 in Material S1). Primer mix D contained 36 primer pairs corresponding to 33 target genes of CoV-HKU1, CoV-229E, RSVB and uncommon respiratory viruses, including parvovirus B19, HHV-1 through HHV-6, polyomavirus, torovirus, CoV-SARS, parechovirus, aichi virus, measles virus, sendai virus, rubella virus, mumps virus and poxvirus and one internal control (NAC1-3) (see Table S4 in Material S1). PCRs were performed in 25-µl volumes containing 1x QIAGEN Multiplex PCR Master Mix (QIAGEN, Hilden, Germany), 500 nM primer UTS, 50 nM each primer from each primer mix, 1,000 copies of cloned plasmid of NAC1, and 2.5µl of the RT product. The application reactions were carried out in a Px2 Thermo Cycler (Thermo Electron Corp, Vantaa, Finland) with initial incubation at 95°C for 15min to activate the HotStar Taq DNA Polymerase; followed by 10 cycles of 94°C for 30s, 55°C for 90s, 72°C for 75s; 10 cycles of 94°C for 30s, 67°C for 90s, 72°C for 75s; 30 cycles of 94°C for 30s, 50°C for 90s, 72°C for 75s; and a final extension at 68°C for 15min.

**Table 3:** Limit of detection of AdV B1 using probit regression analysis^**a**^.

Copies/reaction	Log_10_ copies/reaction	No. of replicates	No. of positive	% positive
1,000	3	10	10	100
500	2.69897	10	10	100
200	2.30103	10	10	100
100	2	10	10	100
50	1.69897	10	10	100
10	1	10	8	80

^a^ Probit regression analysis was calculated by SPSS 17.0 (IBM Corp.), giving a C_95_ value (concentration detectable 95% of the time) of 19.0, which indicate that the limit of detection is about 20 copies/reaction and that samples containing that concentration would be detected 95% of the time.

### Microarray hybridization and processing

The products from the four PCRs were pooled together and subjected to purification and processing, hybridization according to the manufacturer’s recommended protocol (Affymetrix Inc, Santa Clara, CA) [18,19]. The hybridization was performed at 49°C for 16h. The images were scanned and processed as previously described to produce FASTA output files [18,19].

**Table 4:** Limit of detection of CoV-OC43 using probit regression analysis^**a**^.

Copies/reaction	Log_10_ copies/reaction	No. of replicates	No. of positive	% positive
1,000	3	10	10	100
500	2.69897	10	10	100
200	2.30103	10	10	100
100	2	10	10	100
50	1.69897	10	9	90
10	1	10	3	30

^a^ Probit regression analysis was calculated by SPSS 17.0 (IBM Corp.), giving a C_95_ value (concentration detectable 95% of the time) of 59.5, which indicate that the limit of detection is about 60 copies/reaction and that samples containing that concentration would be detected 95% of the time.

### Specificity and sensitivity

The specificity of the assay was investigated with previously confirmed positive specimens infected with 16 common respiratory virus types/subtypes. The target genes of 16 pathogens were amplified with specific primers using the positive samples. Then the products were purified and ligated to pGEM-T vector (Promega, Madison, WI) to construct recombinant plasmids. For RNA viruses, the plasmids amplified by *E. coli* were linearized with *Spe*I and *in vitro* transcribed from the T7 promoter using Ribo-MAX™ large scale RNA Production System T7 (Promega, Madison, WI). The RNA copy number was calculated after measuring the purified RNA concentration by spectrophotometry using Eppendorf Biophotometer (Eppendorf AG, Hamburg, Germany). For DNA viruses, the copy number of amplified plasmids was calculated after measuring the plasmid concentration using Eppendorf Biophotometer (Eppendorf AG, Hamburg, Germany). Ten-fold serial dilutions of these RNA/DNA templates with known copy numbers (10 to 100,000 copies/μl) were used to evaluate the sensitivity of the assay. A panel of six samples with different numbers of copies (1000, 500, 200, 100, 50 and 10) for each of the 16 organisms was prepared. Each sample was tested ten times over five days and the results were analyzed by SPSS 17.0 (IBM Corp.) to calculate the analytical sensitivity [21].

### Assessment of clinical samples

After the evaluation of RPM-IVDC1 assay for known pathogen detection was demonstrated, RPM-IVDC1 assay was used for retrospective diagnoses for the respiratory infections in the setting of CAP. Clinical samples collected from Chaoyang, Shunyi, Fengtai, Dongcheng, and Tongzhou district general hospitals in Beijing from 2011 January to 2011 December, were used to compare the utility of the microarray-based diagnostic to a GeXP-based multiplex RT-PCR assay of respiratory virus detection developed in our laboratory [22]. The specimens were NPAs from patients with clinically documented CAP. Samples were chosen randomly without any test for respiratory pathogens. The lack of culture results and the concern about upper respiratory bacterium contamination make it difficult to properly identify the bacterial pathogens, and so data for these pathogens were not reported.

### RPM-IVDC1 assay data analysis

TessArray Sequence Analysis (TSEQ) software evaluates a "C3 Score" for each RPM-Flu detector tile, as a metric of detected DNA sequence quantity and quality. The C3 Score is the total number of GSEQ-identified nucleotides that appear in runs of three or more consecutive (non-N) base calls, expressed as percentage of the length (nucleotides) of each RPM-Flu detector tile sequence. Relaxed and stringent target pathogen detection and reporting thresholds for the RPM-Flu assay have been statistically and empirically established for all of the resequencing detector tiles at C3 Score >10 or C3 Score ≧20, respectively [16]. In this study, the threshold of C3 Score was increased to ≧50 due to the shortened detector tile sequences and complicated background inherent with clinical samples.

The new method was compared with a multiplex PCR assay [22] for parallel detection of the common sixteen human respiratory virus types/subtypes. For other viruses identified by RPM-IVDC1 as positive but not detected by the reference assay, a conventional PCR and sequencing was performed.

## Results

### Sensitivity and Specificity

**Figure 1:**
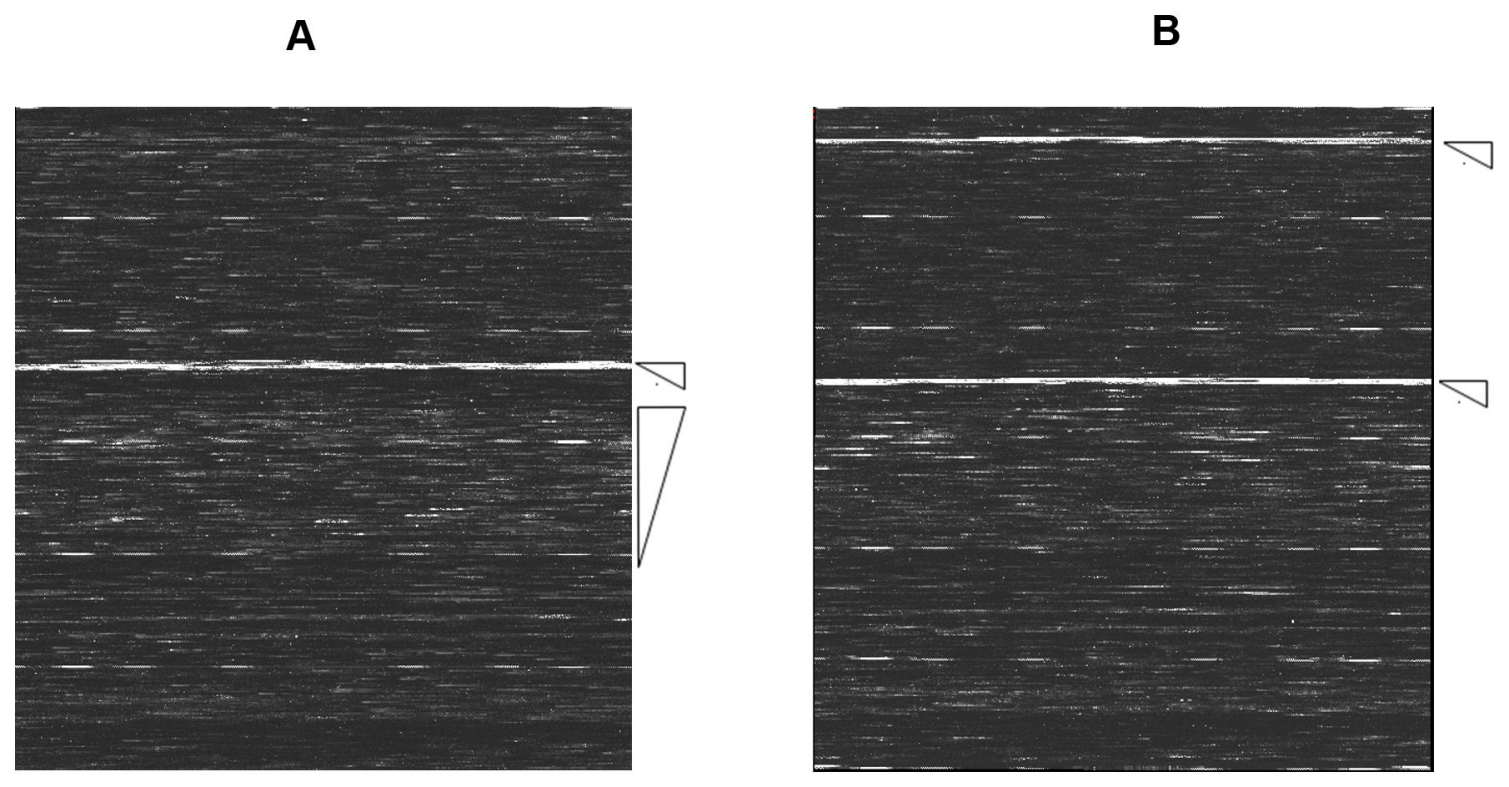
Scanned images of hybridization. (A) Hybridization profile of the influenza type B virus (Upper arrow). Tile region for matrix, (*lower* arrow) noninterfering hybridization profiles of RV and EV. (B) Hybridization profiles of HBoV and CoV-HKU1 (Upper arrow). Tile regions for VP1 and NP1 of HBoV, (*lower* arrow) tile regions for NP and spike of CoV-HKU1.

**Figure 2:**
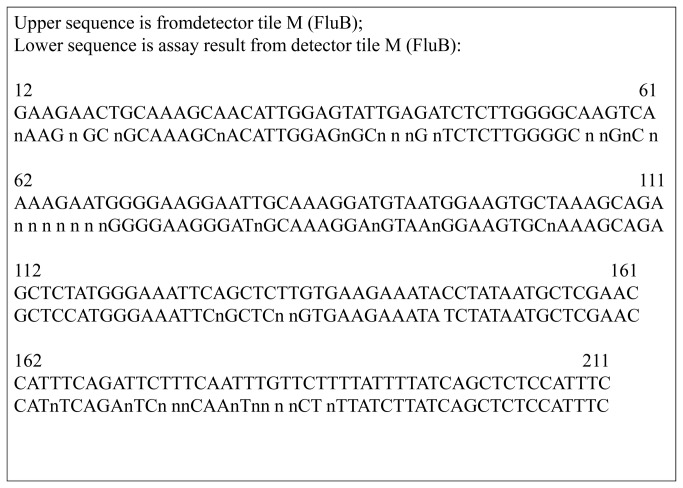
RPM-IVDC1 assay-generated sequences from analysis of an influenza B virus (FluB)-positive sample. A 200-nucleotide segment of sequence generated from RPM-IVDC1 is shown aligned to the corresponding RPM-IVDC1 gene detector tile sequence.

The specificity was confirmed with pre-determined clinical samples (Table 2). Figure 1 show the raw data images generated by the hybridization of influenza type B virus and HBoV/CoV-HKU1 targeted amplicons to the RPM-IVDC1 (the processed sequence data of the images were provided as Material S2 and Material S3). Figure 2 shows the alignment of a 200-nucleotide segment of RPM-IVDC1 assay detected sequence with the corresponding gene detector tile sequence. Noninterfering hybridization, namely hybridization of nontarget amplicons, was detected, resulting in non-specific base calls occurring for a few target probe sets, which was insufficient to cause an incorrect identification [19]. Base calls generated from noninterfering hybridization were mostly observed on RV/EV 5’-UTR and influenza A virus (Flu A) M gene between different types or subtypes. The sequences generated from these noninterfering hybridizations did not cause misidentification, as the noninterfering hybridization was distinguishable from the hybridization of the intended target [19].

The sensitivity of the assay was evaluated using serial 10-fold dilutions of *in vitro* transcribed RNA or DNA clones. The lowest detectable dilutions revealed an individual sensitivity ranging from 10 to 1,000 copies per reaction for 16 virus types/subtypes (data not shown). The limit of detection (LOD) of AdV species B1 and CoV-OC43 using probit regression analysis is shown in Table 3 and Table 4, respectively. Similarly, the detection limits (95% of the time detectable) of other virus types/subtypes were calculated using probit analysis (Table 5), which was comparable to the sensitivity of standard multiplex PCR methods [19].

**Table 5:** Analytical sensitivity of RPM-IVDC1-based detection for individual virus and a pathogen mixture^a^.

Virus	Sample type	detection limit (copies/reaction)^b^	
		individual virus	pathogen mixture
AdV B1	Plasmids	20	20
AdV B2	Plasmids	60	200
AdV C	Plasmids	85	85
AdV DA	Plasmids	85	205
H5N1	cDNA	30	85
CoV-OC43	cDNA	60	570
CoV-229E	cDNA	20	85
CoV-HKU1	cDNA	500	750
RSVA	cDNA	85	270
RSVB	cDNA	25	190
PIV1	cDNA	15	165
PIV3	cDNA	320	175
RV-29	cDNA	20	540
RV-93	cDNA	440	335
RV-25	cDNA	80	350
HBoV	Plasmids	120	85

^a^ The pathogen mixture consisted of 16 nucleic acids from clones and in vitro-transcripts.

^b^ The detection limit was determined by probit analysis using SPSS17.0 (IBM Corp.), representing samples containing this concentration of target organism could be detected 95% of the time. The figures in this table are rounded to the nearest concentration.

### Simultaneous detection and discrimination of respiratory viruses

We further evaluated the ability of RPM-IVDC1 assay to identify multiple pathogens simultaneously by the combination of the 16 common respiratory virus templates. All the cDNA and DNA were mixed at the same concentration to prepare a panel of serial 10-fold dilutions of nucleic acid template. The templates containing 10 to 100,000 gene copies per reaction of 16 pathogens were used to assess the detection sensitivity and specificity for multiple pathogens. The results revealed that this approach allowed simultaneous detection of 16 pathogens at the titer of 1,000 copies/reaction. Probit analysis was performed for limits of detection and calculation of analytic sensitivity (Table 5). The results indicated that RPM-IVDC1–based assay was an effective means of detecting and typing various pathogens with high sensitivity and specificity, when even as many as sixteen pathogens were simultaneously present.

In addition, the ability of RPM-IVDC1 to identify multiple pathogens simultaneously was further determined by diluting only one of two pathogens, representing a situation analogous to actual clinical samples, especially those identified as coinfection. Using the combination of RSVA (10,000 copies/reaction) and AdV B1 (10,000, 1,000, 500, 200, 100, 50, 10 copies/reaction), the probit analysis results showed that RPM-IVDC1 could detect both pathogens to a limit of 22 copies/reaction of AdV B1. In similar experiments, the combinations of AdV B1 and PIV3, RSVA and CoV-229E, RV29 and PIV3, RV29 and CoV-229E also demonstrated that a high concentration target would not outcompete a low-concentration target present in the specimen (data not shown).

**Table 6:** Viruses detected in 110 (age, 0.1-96 years) CAP patients by RPM-IVDC1.

Virus	No. of patients detected by RPM-IVDC1	Co-infection^a^
PIV		
Type 1 (PIV1)	1	1 with RV
Type 2 (PIV2)	2	
Type 3 (PIV3)	4	1 with RSVA, 1with RV
AdV Species B	5	1 with HHV-6
hMPV	1	
RSV		
Type A	4	1 with Flu C and RV
Type B	1	1 with HHV-5
Coronavirus		
CoV-OC43	1	
CoV-NL63	1	
CoV-229E	2	
Influenza virus		
H1N1	3	2 with RV
Type C	1	
HBoV	1	1 with HHV-6
RV		
Species A	21	
Species B	1	
HHV		
Type 1 (HHV-1)	5	1 with RV, 1 with HHV-4 and RV
Type 4 (HHV-4)	7	1 with HHV-6
Type 5 (HHV-5)	1	
Type 6 (HHV-6)	4	
Rubella virus	1	
Measles virus	1	

^a^ In addition to identifying single pathogenic species, another benefit of using RPM-IVDC1 based assay for detection was the ability to detect co-infection and atypical pathogens, such as influenza C virus, rubella virus and measles virus.

### Viral infections

Data on the detection of different viruses by RPM-IVDC1 are shown in Table 6. A viral etiology was found for 53 patients (48%), 12 of whom had more than 1 viral species identified. The most frequently detected virus was RV (22 patients, [20%]), HHV-4 (7 patients, [6.4%]), HHV-1 (5 patients, [4.5%]), AdV (5 patients, [4.5%]), PIV3 (4 patients, [3.6%]), RSVA (4 patients, [3.6%]), HHV-6 (4 patients, [3.6%]). Seven of the 12 patients who had more than 2 viral findings were co-infected with RV and other virus.

**Figure 3:**
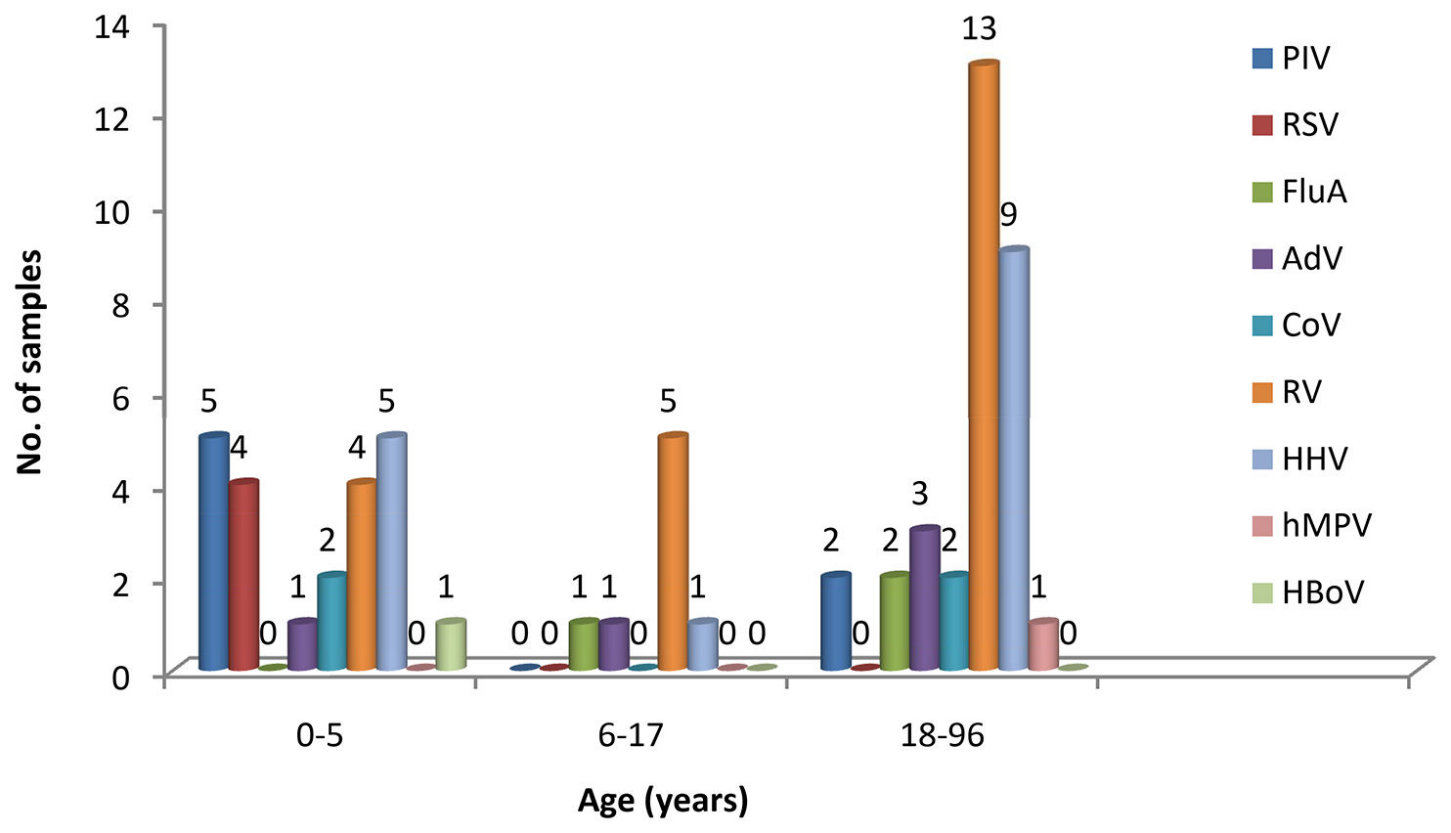
Virus detected in patient groups of different ages by RPM-IVDC1. PIV, parainfluenza virus; RSV, respiratory syncytial virus; FluA, influenza A virus; AdV, adenovirus; CoV, coronavirus; RV, rhinovirus; HHV, human herpesvirus; hMPV, human metapneumovirus; HBoV, human bocavirus.

For PIV1, PIV3, CoV-OC43, CoV-NL63, RSVB and hMPV, the detection result of RPM-IVDC1 completely coincided with the parallel reference assay (Table 7). For RSVA, the RPM detection specificity was 100% but one false-negative was produced. In addition, the RPM assay detected three more cases of seasonal H1N1 infection, two more AdVs, and one more PIV2 than the reference method (Table 8). The improved sensitivity was mainly obtained by the multiple primer pairs which increased the probabilities of amplification and thus achieved better efficiency. The similar situation occurred in human rhinovirus detection where 17 more RVs were detected by RPM. For the atypical pathogens that were not detected by reference assay, the positive specimens identified by RPM were confirmed by PCR and sequencing. For the group of children aged ≤5 years, 16 (72.7%) of 22 patients had viral infections. The rate was higher than among patients aged between 6 to 17 years and the adults group, which were 45% and 39.7%, respectively (Figure 3).

**Table 7:** Comparison of RPM-IVDC1 with GeXP-based PCR assay for detection of PIV1, PIV3, CoV-OC43, CoV-NL63, RSVB, hMPV, HBoV^**a**^.

RPM-IVDC1 results	GeXP-based PCR results													
	PIV1		PIV3		CoV-OC43		CoV-NL63		RSVB		hMPV		HBoV	
	positive	negative	positive	negative	positive	negative	positive	negative	positive	negative	positive	negative	positive	negative
Positive	1	0	4	0	1	0	1	0	1	0	1	0	1	0
Negative	0	109	0	106	0	109	0	109	0	109	0	109	0	109
Sensitivity (%)	100		100		100		100		100		100		100	
Specificity (%)	100		100		100		100		100		100		100	
Agreement (%)	100		100		100		100		100		100		100	

^a^ PIV1, parainfluenza virus type 1; PIV3, parainfluenza virus type 3; CoV-OC43, coronavirus 229E; CoV-NL63, coronavirus NL63; RSVB, respiratory syncytial virus B; hMPV, human metapneumovirus; HBoV, human bocavirus.

**Table 8:** Comparison of RPM-IVDC1 with GeXP-based PCR assay for detection of PIV2, AdV, RSVA, CoV-229E, RV, FluA^**a**^.

RPM-IVDC1 results	GeXP-based PCR results											
	PIV2		AdV		RSVA		CoV-229E		RV		FluA	
	positive	negative	positive	negative	positive	negative	positive	negative	positive	negative	positive	negative
Positive	1	1	3	2	4	0	0	2	5	17	0	3
Negative	0	108	0	105	1	105	0	108	1	87	0	107
Sensitivity (%)	100		100		80		−		83		−	
Specificity (%)	99		98		100		98		84		97	
Agreement (%)	99		98		99		98		85		97	

^a^ PIV2, parainfluenza virus type 2; AdV, adenovirus; RSVA, respiratory syncytial virus A; CoV-229E, coronavirus 229E, RV, rhinovirus; FluA, influenza A virus.

## Discussion

While previous versions of RPM have been validated and showed excellent correlation with the reference assays [16,19,23], more studies are needed to develop and validate novel and improved assays using this technology. More specific amplification strategy and protocols [24] and the design of primers for the amplification of novel targets are needed to allow more widespread clinical adoption of this new tool [19].

Broad-range detection microarrays are used for the direct detection of microbes in complex samples and therefore require nucleic acid amplification to bring the target concentrations to detectable levels [25]. In this study, a targeted multiplex PCR based on temperature-switch PCR (TSP) amplification technology was adopted and a commonly used multiplex PCR reagent was used, which increased assay sensitivity compared to conventional amplification strategy in the case of samples with heavy backgrounds (e.g., human and bacterial genome). The biphasic PCR parameters of the TSP allows a multiplex PCR to be performed under standardized PCR conditions without extensive optimization of each individual PCR assay [22]. Unique buffer in the multiplex kit promotes stable and efficient primer annealing by preventing nonspecific primers from annealing to the template and increasing the local concentration of primers at the template and allows efficient extension. This improvement therefore made it easy to group the pathogens into subsets for efficient amplification without much optimization, which allowed stable performance when new primers were added into primer mix or when the primers were changed [23].

The combination of a targeted multiplex PCR and microarray hybridization should allow good analytical sensitivity in the case of samples with heavy backgrounds. The ability of differentiating virus types or subtypes relies on the sequences obtained from the targeted amplicons hybridization to the detector tiles. Thus, although a completely novel respiratory pathogen would not be captured by this approach, a mutant virus or an uncommon pathogen could be captured. Another benefit of association of targeted multiplex PCR and resequencing microarray is the simultaneous detection and characterization of several viruses, which allows a significant time-savings compared to conventional assays or sequencing, and could be an important element in improving emergency response capacity.

Another improvement of RPM-IVDC1 versus standard detection tools is the more comprehensive coverage of target organisms it allows. In contrast to RPM-Flu3.1, about 30% of which is dedicated to targeting all 16 *HA* and 9 *NA* alleles of avian influenza A viruses [26], the new resequencing array design of RPM-IVDC1 allows more coverage of respiratory viral pathogens. The two main changes of the new microarray design compared to RPM-Flu3.1 were the length of the detector tiles and the primers selected for amplification of the chosen targets. The shortening of detector tiles allows an increase in the number of probe sets to be placed on the microarray, and therefore almost all common respiratory viral pathogens could be detected by RPM-IVDC1 in a single assay. Some atypical respiratory pathogens (e.g., measles virus, rubella virus and influenza C virus) and non-respiratory virus (e.g., HHV) could be detected by RPM-IVDC1, but the probes corresponding to these are not present in RPM-Flu3.1. Furthermore, the new technology, by using one or more pairs of primers designed for amplification of conserved regions, allows rapid and efficient strain-level identification for some pathogens, which may be critical in outbreak investigations.

In addition to improvement of the amplification method and primer design, new internal controls were used in RPM-IVDC1. Two *Arabidopsis thaliana* genes, NAC1 and TIM, were selected as internal controls as these plant genes would be unlikely to occur naturally in clinical samples [19]. Comparing with RPM-Flu3.1, new overlapped detector tiles for these controls were tiled on the new microarray and new primers were designed to amplify shorter detector sequences, which could improve the hybridization efficiency. The results showed the base call rates of TIM and NAC1 could reach over 90% without interference with the detection of microbial agents. As a result of new internal controls, an unqualified chip without any base called may be found and thus avoiding false-negative results.

The number of targets that needs to be validated depends on the intended purpose of the study. Control strains of febrile respiratory illness (FRI)-causing pathogens [18,19] and different reference strains of influenza A virus [16] were used to assess the performance of RPM v.1 and RPM-Flu3.1, respectively. In this study, sixteen single-virus infected reference specimens were used to evaluate the new microarray. The results showed excellent specificity of the new microarray for type- or strain-level identification of some reference samples. As the new resequencing microarray was designed to be smaller than previous versions to increase the hybridization efficiency, the lengths of the detector tiles of RPM-IVDC1 were much shorter and the numbers of the tiles were relatively limited. As a consequence, the conserved gene sequences obtained from detector tiles of some pathogens could differentiate samples at a strain level for only a few viruses (hMPV, CoV-OC43, RSVA, RSVB, PIV1, PIV3). In addition, some viruses have many serotypes, thus for these, the identification could only be achieved at species level (AdV, RV).

The results of RPM-IVDC1 showed excellent correlation with the reference assay for the detection of common respiratory pathogens with only one false-negative of RSVA. But since one additional occurrence of PIV2 infection, two AdVs, sixteen RVs, three FluAs and two CoV-229Es were detected by the new assay, the increased sensitivity of the assay compared with reference method is likely attributable to the use of multiple conserved primer pairs targeting specific pathogens and the high efficiency of the assay. This was particularly noticeable in the RV identification, where only 5 of the 22 RPM-identified RV infections were detected by the reference assay. The use of twelve RV type-specific primer pairs in the RPM primer mix compared to only one pair in the reference assay [22] likely enables improved detection of RV.

The detection rate of common viruses was consistent with previous research [9,27]. The most commonly detected virus in children aged under 5 years was RSV; 4 out of the 5 detected RSVs was type A. The most common etiologic agent in the adult group was RV, of which 21 out of 22 infections (95.5%) clustered within type A. In an analysis of the 68 detection-positive patients with complete clinical data, no significant seasonal predominance was observed for the total numbers of virus detected compared with previous study [28]. Neither the obvious difference of detection rate was observed among the groups with various disease severities due to the broad detection extent of the aetiological or anetiological virus and the small sample size.

As a result of the broad range of respiratory viruses identifiable with this assay, some atypical pathogens were detected using the new microarray. One instance of measles virus, one rubella virus and one influenza type C infection were found in patients aged 13 years, 13 years and 1 month, respectively. Rubella virus is the pathogenic agent of rubella, which is a common childhood infection but could affect anyone of any age, with the possible complications of fatal pneumonia [29]. As the causative agent of measles, the presence of the measles virus may also lead to serious problems, including measles pneumonia, which is a life-threatening complication in children [30]. Influenza C virus, which is sometimes present in patients with a clinical diagnosis of pneumonia, could cause a variety of respiratory illnesses that cannot be clinically differentiated from those caused by other viruses [31]. HHV is ubiquitous in humans, causing lifelong infection. After primary infection, which usually occurs during childhood, the virus remains latent in the ganglia of sensory neurons. Systemic stimuli including fever due to bacterial or viral infection can cause reaction of the virus [32] and potentially clinical disease, resulting in active secretion in the throat. Although there are few reports of symptomatic pulmonary involvement directly attributable to HHV infection [33], the presence of HHV in the throat is still a highly significant and independent risk factor for the development of lower respiratory tract infections with HHV [32,34]. Sequences of HHV-1 through HHV-6 were tiled on RPM-IVDC1 and 5 instances of HHV-1, 7 HHV-4 (EBV), 1 HHV-5 (CMV), 4 HHV-6 infections were detected using the RPM method, which was subsequently confirmed by PCR and sequencing. Of the 15 patients infected with HHV, 2 were co-infected with HHV-6 and HHV-4 and another one was co-infected with HHV-1 and HHV-4. HHV-6 virus, which causes roseola and fever after the primary infection, was identified in three children with ages under 5 and one aged 8. All HHV-1-positive samples and 5 (71.4%) of 7 HHV-4-positive specimens were from adult patients; the frequency of HHV in the NPAs (13%) of adult patients were lower than found in a previous study (22%) [32], which might be due to the difference between the sample characteristics. Although it is not possible in a retrospective study, and without the concomitant detection of bacterial pathogens, to state that these atypical pathogens were causative agents, the results of this study show the capability of RPM-IVDC1 for the detection of atypical pathogens and the potential for diagnosing infectious syndromes manifesting by similar symptoms but caused by diverse etiological agents.

The accurate identification of multiple respiratory tract viral pathogens using RPM-IVDC1 in this study demonstrates that this novel assay could improve the response capacity to epidemic outbreak and be significant tool for public health. However, the false-negative detection for RSVA should be addressed prior to clinical adoption. The capability of this assay for the simultaneous detection of a broad-spectrum respiratory tract viruses should also be further validated when considering the application of this new RPM towards outbreak investigations or epidemic surveillance.

## Supporting Information

Material S1 **The viruses targeted by RPM-IVDC1.** The targeted viruses and corresponding genes are listed in Table S1-Table S4, which lists the primers in primer mix A, B, C and D used for multiplex PCR, respectively.
(DOC)

Material S2 **The raw sequence data generated by the scanned image of influenza type B virus.** The raw sequence data were generated after processing the scanned image with TessArray Sequence Analysis (TSEQ) software. The base of the sequence was written with “n” when there was no amplicon hybridized to corresponding detector tile.
(TXT)

Material S3 **The raw sequence data generated by the scanned image of HBoV and CoV-HKU1 virus.** The raw sequence data were generated after processing the scanned image with TessArray Sequence Analysis (TSEQ) software.
(TXT)
